# Sir R. J. Collie on a State Medical Service

**Published:** 1914-01-17

**Authors:** 


### The Hospital

**The Workers' Newspaper of Administrative Medicine and Institutional Life, Administration, National Insurance and Health.**

No. 1438, Vol. LV. SATURDAY, JANUARY 17, 1914. PRICE ONE PENNY

### SIR R. J. COLLIE ON A STATE MEDICAL SERVICE.

Those who maintained that the National Insurance Act was partly intended as a wedge to make way for the complete socialisation of the medical profession by means of a State Medical Service have soon been justified of their opinions. Already Sir John Collie, the medical referee to the London County Council and the Metropolitan Water Board, and one of those on whom the framers of the Act are believed to have relied very largely for advice and information, has come boldly into the open with a public lecture in support of such a measure. Sir John sees clearly enough what has been insisted on from the first in The Hospital—namely, that the Insurance Act omits altogether to provide medical benefit for cases of serious illness and of accident, and so fails hopelessly as a constructive piece of statesmanship. He further admits, apparently, that many panel practitioners do not render efficiently to their patients even that modicum of elementary attention which is required of them; and he also recognises the breakdown of the Act in respect of persons away from home.

He proposes to abolish these reproaches, and also to remedy the omission of the dependants of insured persons from the scope of the Insurance Act, by a comprehensive scheme of reform whereby the whole of the less affluent classes shall receive medical attendance through the intervention of the State; such attendance to be in reality, not in pretence as under the panel system, of first-rate quality and to include treatment by surgeons, specialists of all kinds, and general practitioners. The report in the *Times* of Sir John's lecture at the Central Hall, Westminster, on January 12, does not make it perfectly certain whether or not he advocates also nationalisation of the voluntary hospitals; but presumably this is what he intends, for in enumerating the shortcomings of the present Act he mentioned that for all serious diseases the insured classes are still dependent upon charitable institutions.

Those who profess to find nothing but benefit to the insured and the medical profession in the present panel system will find in this lecture some very frank criticisms of the working of National Insurance. Those, on the other hand, who object to the Act because it is injurious to private practice or because it penalises and tyrannises so many of the insured, will find little comfort in plans for perpetuating and extending contract practice on the vast scale now suggested. One point Sir John seems to have overlooked. If the panel practitioners of to-day render such poor service, in the aggregate, as he says they do, where is he to find men of the right sort to staff a vastly greater medical service, and how is the money to be found to pay them? He states that the panel system is extravagant, and that his plan would cost less. But he gives no proofs of this; and his scheme includes regular holidays, pensions, time for study, and freedom from overwork. With all respect for Sir John, these phantasies savour of the political cheapjack, promising the millennium at positively no expense at all.

Meanwhile, there are two parties to the argument whose voices are yet to be heard: to wit, the public and the doctors. The latter he dismisses cavalierly enough. Those in middle-class practice may suffer to some extent, he airily remarks; but seemingly he expects no serious resistance from them. Appointments to all "senior positions," he proposes, shall be by examination, as in the Army and Navy medical services. This suggestion seems to us visionary and impracticable: there is no true comparison between general practice and the duties of naval and military officers. Possible objections from the insured classes are not even discussed; though, considering the wide dissatisfaction with the panel system, the probability that there would be great objections to an extension of the principle is by no means remote.

However, the thanks of all those interested in the care of the sick are due to Sir R. J. Collie for his outspokenness. We know now how matters stand in regard to the future of voluntary institutions in this country. It is clear as daylight that the advanced wing of the Ministerial party, always coquetting with *doctrinaire* Socialist ideals, is seriously contemplating an attempt to nationalise the hospitals, and to socialise the medical profession and about 99 per cent. of the population of the kingdom. This avowal puts an end to speculations based upon observed facts and tendencies. That the Chancellor of the Exchequer approves of the outlines of the scheme put forward at Westminster we are, indeed, not actually informed. But it is so extremely probable that it can be assumed with the barest and minimal chances of error. It will be of interest to see whether he will succeed in forcing so daring a project on the staider sections of his party; but meanwhile the policy disclosed by Sir John Collie is of greater importance as a clear challenge to those who are not convinced of the utility of a State Medical Service. The matter will have to be threshed out and decided by public opinion; but public opinion requires to be led, and it is evident that certain sections of political thought are preparing to lead it, if they can. It therefore behoves the opponents of such a policy to bestir themselves likewise, or to suffer the rebuff of the unready. A plain warning has been given, which there is no excuse for neglecting.

